# Combination of Cytokine-Induced Killer Cells and Programmed Cell Death-1 Blockade Works Synergistically to Enhance Therapeutic Efficacy in Metastatic Renal Cell Carcinoma and Non-Small Cell Lung Cancer

**DOI:** 10.3389/fimmu.2018.01513

**Published:** 2018-07-05

**Authors:** Zibing Wang, Xiaoli Liu, Brian Till, Miaomiao Sun, Xiang Li, Quanli Gao

**Affiliations:** ^1^Affiliated Hospital of Zhengzhou University, Henan Cancer Hospital, Zhengzhou, China; ^2^Fred Hutchinson Cancer Research Center, Seattle, WA, United States

**Keywords:** cytokine-induced killer cells, programmed cell death-1, immunotherapy, lung cancer, renal cell carcinoma

## Abstract

**Introduction:**

Programmed cell death-1 (PD-1) inhibition therapy has changed the treatment paradigm of metastatic renal cell carcinoma (MRCC) and non-small cell lung cancer (NSCLC). However, attempts to use the drug as a single agent have achieved only limited clinical success. To further enhance the clinical benefits of monotherapy, combination therapies will likely be necessary. Cytokine-induced killer (CIK) cells are a heterogeneous subset of *ex vivo* expanded T lymphocytes that have been shown to prolong the survival of cancer patients. We are conducting a study to evaluate the efficacy of PD-1 inhibitor in combination with CIK cells in relapsed/refractory MRCC and NSCLC and to analyze potential biomarkers to predict which patients will benefit most from the combined therapy.

**Case presentation:**

The results of two patients treated in an ongoing clinical trial for MRCC and NSCLC are described here. The tumor biopsy from Patient 1 exhibited moderate CD3^+^ T cell infiltration, but no PD-1 or PD-L1 expression. The tumor cells from Patient 2 strongly expressed PD-L1, and there was extensive tumor infiltration by CD3^+^ T cells; however, no PD-1 staining was seen. Non-synonymous single nucleotide variant (nsSNVs), along with higher indel mutations, in Patient 1 and nsSNVs along with higher tumor mutation burden in Patient 2 correlate with tumor-infiltrating CD3^+^ lymphocyte density. Patient 1 achieved a complete response, and Patient 2 achieved a near-complete response.

**Conclusion:**

A PD-1 inhibitor in combination with CIK cells led to potent antitumor activity in MRCC and NSCLC; CD3^+^ T cell infiltration in baseline tumor biopsies is a potential predictive biomarker. This approach is being further investigated in an ongoing phase I trial.

## Introduction

Although blocking of programmed cell death-1 (PD-1) alone has shown great promise in the treatment of advanced solid tumors, combined immunotherapies will be required to efficiently treat a wider range of cancer patients (1). Cytokine-induced killer (CIK) cells are a heterogeneous subset of *ex vivo* expanded T lymphocytes, comprising CD3^+^CD56^+^ cells, CD3^−^CD56^+^ natural killer (NK) cells, and CD3^+^CD56^−^ cytotoxic T cells (2). CIK cells have been shown to prolong the survival of patients in metastatic renal cell carcinoma (MRCC) and non-small cell lung cancer (NSCLC) with minimal side effects (3, 4). Here, we show two representative cases from an ongoing clinical trial of patients with MRCC and NSCLC that were successfully treated with the combination therapy. Written informed consent was obtained for the use of anti-PD-1 antibodies and CIK cells in both patients.

## Case Presentation

**Figure 1:**
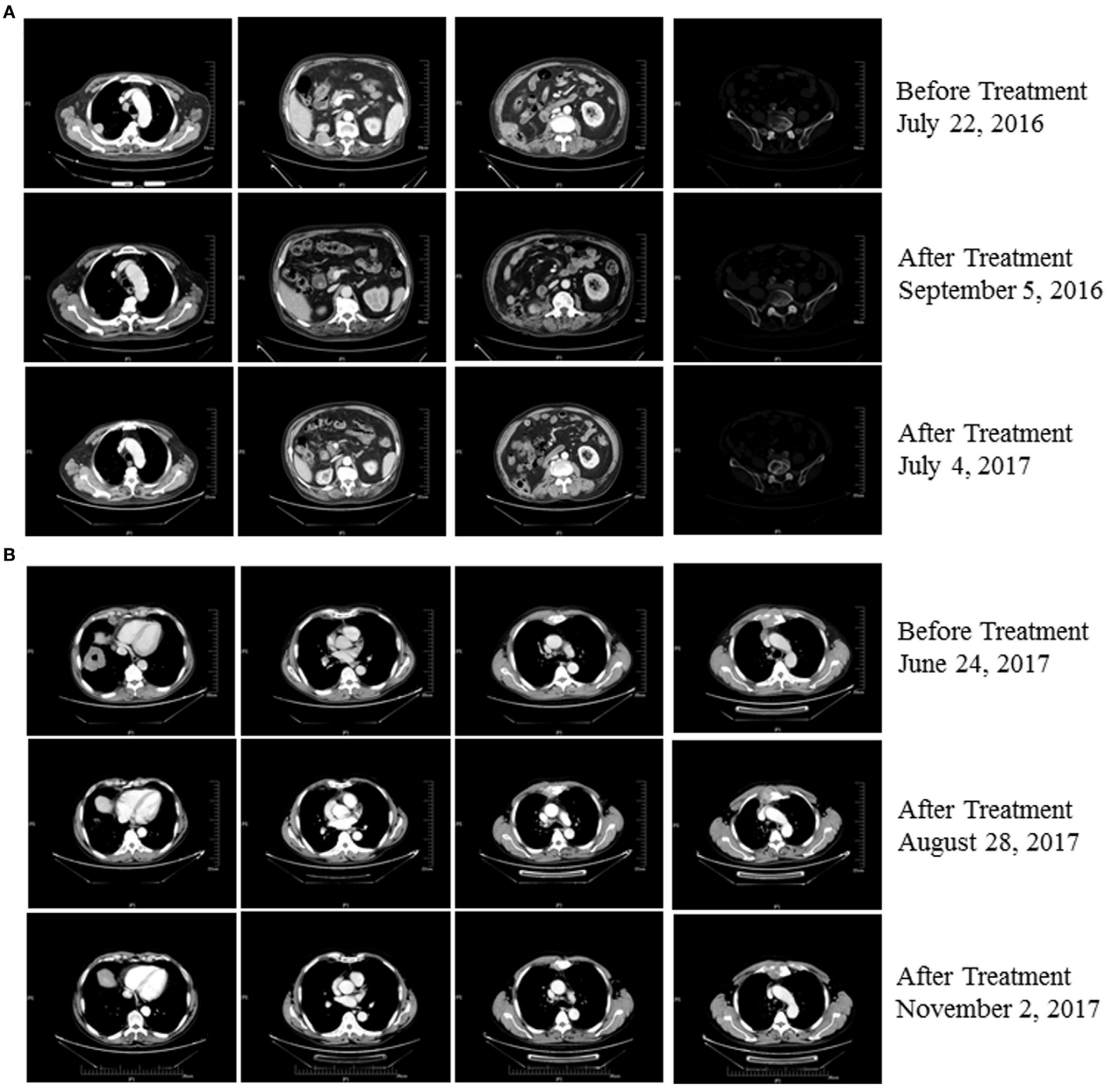
**(A)** Patient 1 exhibited a complete response after three cycles of treatment with pembrolizumab plus cytokine-induced killer (CIK) cell transfer and continues to be in remission 537 days posttreatment (as of 12/02/2017). **(B)** Patient 2 exhibited a partial response after two cycles of treatment with pembrolizumab plus CIK cell transfer and continues to be in remission on day 185 after treatment (as of 12/02/2017).

An 80-year-old man (Patient 1) received a diagnosis of clear cell carcinoma of the right kidney after partial nephrectomy. One year later, computed tomography showed a right lumbar mass, and surgical specimens revealed clear cell carcinoma. The patient subsequently underwent afatinib treatment. However, therapy was discontinued as a result of intolerable adverse effects. Eighteen months later, PET-CT showed multiple metastases, including to the right pleural tubercle, thoracic vertebra, lumbar vertebra, left ilium, and humerus. In consultation with the radiation therapist, he received stereotactic radiotherapy to the right lung; however, a new left upper gingival soft tissue mass was found during the radiotherapy, and tumor metastasis was confirmed by biopsy. He was then treated with pembrolizumab combined with CIK cell transfer. CIK cells were prepared as described previously (3). Briefly, peripheral blood mononuclear cells (PBMC) were separated and cultured under sterile conditions in 1640 medium containing anti-CD3 monoclonal antibody, interferon γ, interleukin-2, and RetroNectin. After culturing the cells for 10–14 days, a target dose of about 6 × 10^9^ CIK cells with over 95% viability was obtained and tested for biological contaminants. Cells were then prepared in sodium chloride solution containing 2% albumin before transfusion. He achieved a complete response following treatment with four cycles of pembrolizumab combined with eight cycles of CIK cell transfer (Table S1 in Supplementary Material) and continues to be in remission on day 537 of the first dose of pembrolizumab treatment (Figure 1A). The patient had gingivitis after first cycle of pembrolizumab and pneumonia after second cycle of pembrolizumab, for which he received systematic antibiotic treatment. He did not receive glucocorticoids or other immunomodulating agents during his treatment with pembrolizumab and CIK cells.

A 63-year-old man (Patient 2) received a diagnosis of squamous cell carcinoma after biopsy of a right lower lobe lung mass. CT scans showed that this patient had developed multiple metastases, including mediastinal, right hilar, and anterior superior phrenic lymph nodes, and also to the sternum. His disease progressed following first-line platinum-based doublet chemotherapy and second-line S-1, a second-generation oral fluoropyrimidine composed of tegafur, gimeracil, and oteracil (5). He was then treated with pembrolizumab combined with CIK cell transfer as third-line therapy. Follow-up imaging showed a near-complete response after treatment with eight cycles of pembrolizumab in combination with seven cycles of CIK cell transfer (Table S1 in Supplementary Material), and the patient continues to be in remission 185 days posttreatment (Figure 1B). No adverse events occurred during treatment in this patient.

**Figure 2:**
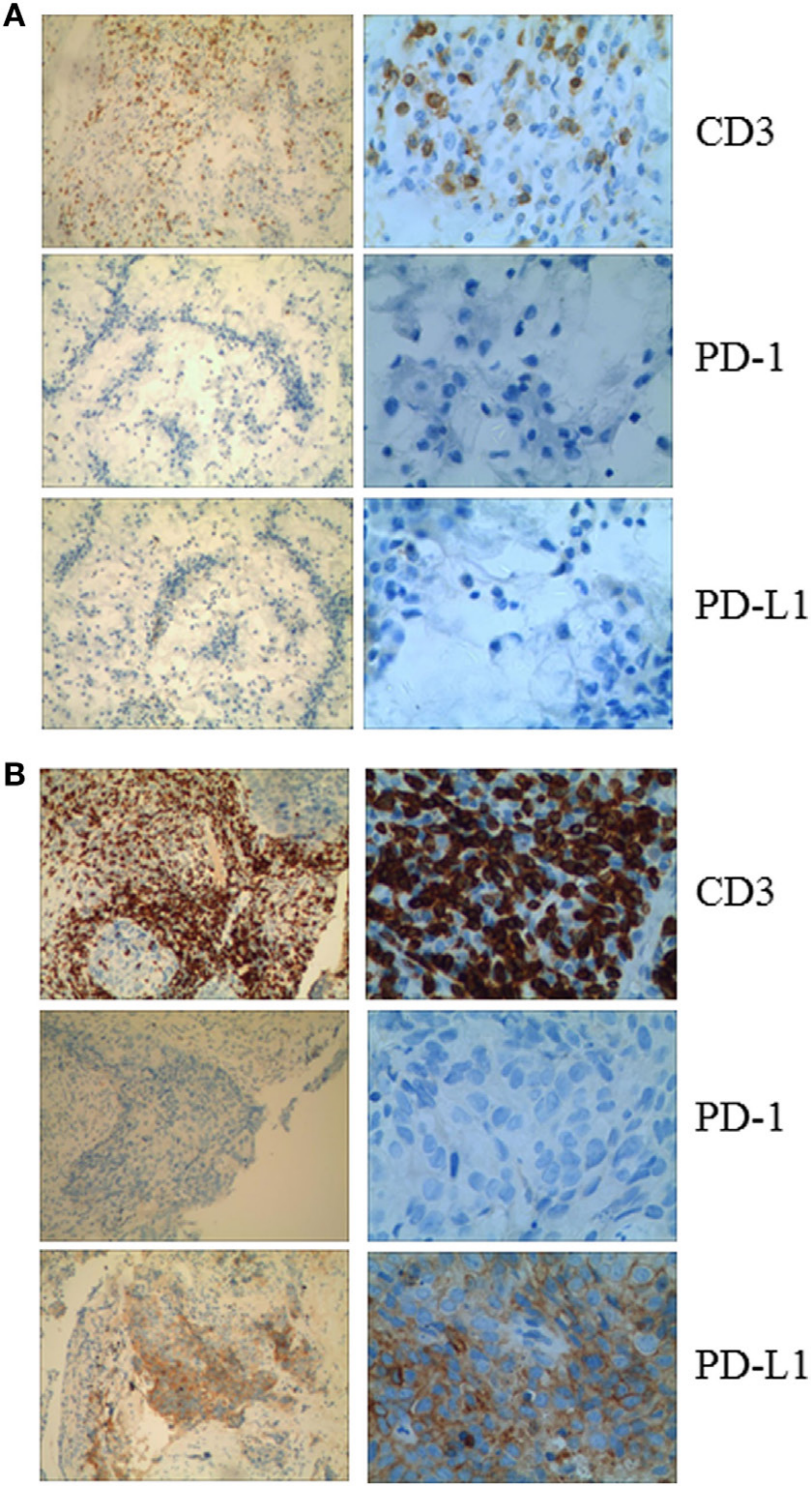
**(A)** Immunohistochemical analysis in Patient 1 shows no membranous expression of programmed cell death-1 or PD-L1 in the tumor cells and the small background cells surrounding the malignant tumor cells. CD3^+^ cells are observed among the tumor cells. **(B)** Immunohistochemical analysis of a pretreatment tumor in Patient 2 shows focal membranous expression of PD-L1 in the tumor cells, with additional expression seen on the small background cells surrounding the malignant tumor cells. Abundant CD3^+^ cells are identiﬁed among the tumor cells (magniﬁcation: left panel, 10×; right panel, 40×).

**Figure 3:**
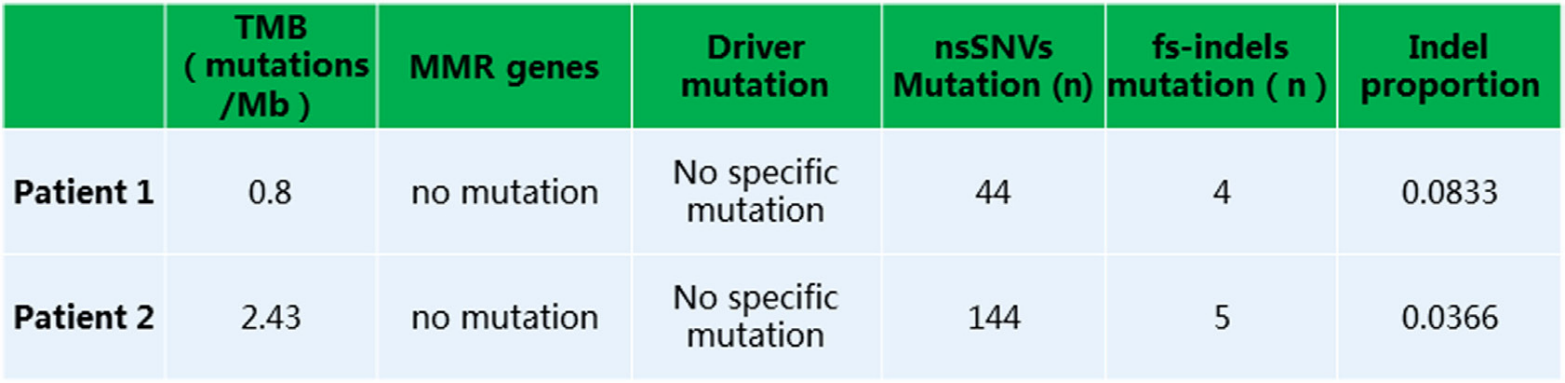
Whole exome sequencing analysis revealed a low tumor mutation burden, lack of mutation in MMR or driver genes, and presence of non-synonymous single nucleotide variants or fs-indels.

Given that PD-1 pathway blockade induces a clinical response in subsets of cancer patients, defining biomarkers that predict therapeutic effects and adverse events is crucial. Recent studies have suggested an association between clinical responses and several promising biomarkers, including PD-1, PD-L1, CD3, tumor mutation burden (TMB), deficient DNA mismatch repair status (dMMR), and insertions and deletions (indels) (6). To examine the relationship between PD-1, PD-L1, or CD3 expression in the tumors and antitumor responses elicited by anti-PD-1 antibodies combined with CIK cell transfer, we evaluated pretreatment tumor biopsies from both patients using immunohistochemistry and found that the tumor biopsy from Patient 1 exhibited moderate CD3^+^ T cell infiltration, but no PD-1 or PD-L1 expression (Figure 2A). The tumor cells from Patient 2 strongly expressed PD-L1, and there was extensive tumor infiltration by CD3^+^ T cells; however, no PD-1 staining was seen (Figure 2B). It has been reported that frameshift indels are more prevalent in renal cell carcinoma compared with other tumor types and are associated with enhanced T-cell activation (7). Across all tumor types, clear cell renal carcinoma was found to have the highest proportion of coding indels, 0.12, a 2.4-fold increase when compared with the pan-cancer average, 0.05 (7). We analyzed the whole-exome sequences of pretreatment tumors and patient-matched blood samples. We observed an indel proportion value of 0.08 and an indel count of 4 in patient 1, while these values were 0.04 and 5, respectively, in patient 2. We also found that tumors from both patients harbored non-synonymous single nucleotide variant (nsSNV) mutations. The TMB in patient 2 was higher than that in patient 1 (2.4 versus 0.8/Mb, respectively); however, no mutations in MMR-associated genes occurred in either patient (Figure 3). Based on these data, we hypothesize that nsSNVs, along with higher indel mutations, in patient 1 with renal cell carcinoma and nsSNVs along with higher TMB in patient 2 with lung cancer correlate with tumor-infiltrating CD3^+^ lymphocyte density. Therefore, CD3^+^ T cell infiltration in baseline tumor biopsies may be a potential biomarker to predict which patients will benefit most from PD-1 blockade in combination with CIK cell therapy, although this will need to be confirmed in larger numbers of patients.

## Discussion

Immune checkpoint inhibitors, specifically PD-1-directed agents, have been approved for the treatment of advanced MRCC and NSCLC. Landmark trials have demonstrated a survival advantage with these agents compared to standard-of-care first-line and second-line therapies, along with greater durability of response in some patients. However, the majority of patients still do not respond to PD-1 inhibitors. Therefore, new therapies and novel combinations will be necessary.

One such novel therapy might be to combine adoptive cell transfer with PD-1 inhibitors. Among various adoptive lymphocyte therapies, CIK cells have a particularly advantageous profile as these cells are easily obtained, have a high proliferation rate, and exhibit a potent antitumor activity (8). In previous studies, we showed that CIK cells alone induced regression of MRCC with no major side effects after repeated transfer (3), and the efficacy was significantly improved when CIK cells were combined with chemotherapy (9). Similar results were reproduced in advanced pancreatic cancer (9, 10). Based on these promising findings, we sought to investigate the potential clinical efficacy of the combination of PD-1 inhibitor with CIK cells in MRCC and NSCLC.

Programmed cell death-1 pathway blockade in combination with CIK cell transfusion elicited durable clinical responses in these patients with a reasonable toxicity profile. The precise mechanisms responsible for the observed antitumor responses are not clear, but we speculate that the several potential factors may play a role.

First, anti-PD-1 therapy may increase the absolute number of effector T cells in the tumor microenvironment. It is known that anti-PD-1 therapy largely relies on efficient T cell infiltration into tumors and effector T cell function in the tumor microenvironment. Most CIK cells are CD3^+^CD56^−^ T cells and are thought to have the ability to migrate to tumor sites, based on the observation that a sustained strong bioluminescent imaging signal from the tumor site was still evident 3 weeks after adoptive transfer of luciferase-positive CIK cells in murine model of A20 B-cell lymphoma (11). We hypothesize that in the clinical setting, adoptively transferred CIK cells encounter anti-PD-1 antibodies, neutralizing an inhibitory signal generated by binding of PD-1 on these cells to anti-PD-1 antibodies. This assumption is supported by our finding that percentage of the PD-1^+^ subpopulation among circulating CD3^+^ T cells substantially decreased after treatment compared to baseline levels in both patients (Tables S2 and S3 in Supplementary Material). However, it is unclear whether CIK cell activity is boosted in the periphery by anti-PD-1 antibodies, and then they migrate into the tumor and kill tumor cells; or if the cells are already infiltrating the tumors but become exhausted or inhibited by PD-1 signaling, and their activity is subsequently restored by the PD-1 blockade.

Second, CIK cells may have a direct cytotoxic effect against tumor cells. It has been shown that during CIK cell expansion, NKG2D is upregulated, which is an activating receptor expressed on NK cells and plays a crucial role in tumor cell killing (11, 12). Interestingly, a recent study reported an increase of an unusual population of peripheral blood cells expressing CD56 in patients with regressing melanoma following PD-1 blockade treatment (13). Thus, an increase in the total number of CD56^+^ cells might contribute to the efficacy of the combination therapy of CIK cells and PD-1 blockade. To assess this, we measured CD56^+^ NK cells in the PBMC before and at various time points after treatment and found that this cell population approximately doubled after treatment in both patients (Tables S2 and S3 in Supplementary Material).

Third, CIK cells secrete high amounts of IFN-γ. Thus, in addition to increasing the number of T lymphocytes in the tumor microenvironment, the provision of cytokine help to existing tumor-infiltrating T cells may augment their function and overcome mechanisms of immune escape such as MHC downregulation, ultimately shifting the balance in the tumor microenvironment in favor of an antitumor response.

The feasibility of generating CIK cells is a potential limitation of the combined treatment. The laboratories producing CIK cells must be specialized and have highly trained personnel. Additionally, there are differences in the techniques used by different laboratories in manufacturing CIK cells. One difference between ours and that reported by others was that we added RetroNectin to the traditional CIK cell culture system. The CIK cells prepared using this method secreted more Th1 cell-associated cytokines compared to traditional CIK cells (14). However, whether there is a difference in efficacy between traditional CIK cells and RetroNectin-activated CIK cells when they are combined with PD-1 inhibitor is unclear.

## Concluding Remarks

Programmed cell death-1 blockade in combination with CIK cells showed promising clinical responses in two patients with MRCC and NSCLC and is being further investigated in an ongoing clinical trial.

## Ethics Statement

No investigations or interventions were performed outside routine clinical care for these patients.

## Author Contributions

ZW and QG have designed the paper; MS and XL provided technical or material support; ZW, XL, and BT wrote, reviewed, and revised the paper; all the coauthors revised paper critically and gave final approval of this version for publishing.

## Conflict of Interest Statement

The authors declare that the research was conducted in the absence of any commercial or financial relationships that could be construed as a potential conflict of interest.

## Abbreviations

PD-1, programmed cell death-1; MRCC, renal cell carcinoma; NSCLC, non-small cell lung cancer; CIK, cytokine-induced killer; NK, natural killer; TMB, tumor mutation burden; dMME, deficient DNA mismatch repair status; indels, insertions and deletions; nsSNV, non-synonymous single nucleotide variant; PBMCs, peripheral blood mononuclear cells.
