# Supplementary material for: Combination of Cytokine-Induced Killer Cells and Programmed Cell Death-1 Blockade Works Synergistically to Enhance Therapeutic Efficacy in Metastatic Renal Cell Carcinoma and Non-Small Cell Lung Cancer
Source: Front Immunol. 2018 Jul 5;9:1513. doi: 10.3389/fimmu.2018.01513 (PMC6041387; doi:10.3389/fimmu.2018.01513)
Supplement: Supplementary file 1 [file table_1.PDF]

Table 1. Time of pembrolizumab and CIK cell use in patients

**Patient 1**		**Patient 2**	
**Pembrolizumab**	**CIK cells**	**Pembrolizumab**	**CIK cells**
Day 0	Day 29	Day 0	Day 16
Day 16	Day 46	Day 30	Day 44
Day 67	Day 63	Day 50	Day 65
Day 100	Day 79	Day 71	Day 86
	Day 97	Day 92	Day 107
	Day 117	Day 113	Day 128
	Day 147	Day 139	Day 153
	Day 225	Day 159	
