# Supplementary material for: Combination of Cytokine-Induced Killer Cells and Programmed Cell Death-1 Blockade Works Synergistically to Enhance Therapeutic Efficacy in Metastatic Renal Cell Carcinoma and Non-Small Cell Lung Cancer
Source: Front Immunol. 2018 Jul 5;9:1513. doi: 10.3389/fimmu.2018.01513 (PMC6041387; doi:10.3389/fimmu.2018.01513)
Supplement: Supplementary file 2 [file table_2.PDF]

Table 2. Percentage of subpopulation in the PBMC in patient 1

Cell types	Percentage at different days				
	Day 0	Day 39	Day 66	Day 147	Day 211
Total $CD3^+$ T lymphocyte	73.5	80.1	81.4	85.4	87.8
PD-1^+ subpopulation in $CD3^+$ T cells	8.8	0.0	1.1	1.3	1.9
$CD56^+$ NK cells	8.1	17.2	15.7	9.6	6.1
Tregs	8.8	14.5	16.8	6.9	12.2
MDSCs	0.2	0.1	0.6	0.3	0.4
