# Supplementary material for: Combination of Cytokine-Induced Killer Cells and Programmed Cell Death-1 Blockade Works Synergistically to Enhance Therapeutic Efficacy in Metastatic Renal Cell Carcinoma and Non-Small Cell Lung Cancer
Source: Front Immunol. 2018 Jul 5;9:1513. doi: 10.3389/fimmu.2018.01513 (PMC6041387; doi:10.3389/fimmu.2018.01513)
Supplement: Supplementary file 3 [file table_3.PDF]

Table 3. Percentage of subpopulation in the PBMC in patient 2

Cell types	Percentage at different days			
	Day 0	Day 21	Day 90	Day 160
Total $CD3^+$ T lymphocyte	65.0	50.7	52.6	54.4
PD-1^+ subpopulation in $CD3^+$ T cells	8.1	1.4	2.0	0.3
$CD56^+$ NK cells	19.4	36.9	39.9	40.3
Tregs	11.2	10.8	12.0	10.9
MDSCs	0.3	0.5	0.7	0.5
